# Principles of self-organization in biological pathways: a hypothesis on the autogenous association of alpha-synuclein

**DOI:** 10.1093/nar/gkt794

**Published:** 2013-09-03

**Authors:** Andreas Zanzoni, Domenica Marchese, Federico Agostini, Benedetta Bolognesi, Davide Cirillo, Maria Botta-Orfila, Carmen Maria Livi, Silvia Rodriguez-Mulero, Gian Gaetano Tartaglia

**Affiliations:** ^1^Gene Function and Evolution, Bioinformatics and Genomics, Centre for Genomic Regulation (CRG), 08003 Barcelona, Spain and ^2^Universitat Pompeu Fabra (UPF), 08003 Barcelona, Spain

## Abstract

Previous evidence indicates that a number of proteins are able to interact with cognate mRNAs. These autogenous associations represent important regulatory mechanisms that control gene expression at the translational level. Using the catRAPID approach to predict the propensity of proteins to bind to RNA, we investigated the occurrence of autogenous associations in the human proteome. Our algorithm correctly identified binding sites in well-known cases such as thymidylate synthase, tumor suppressor P53, synaptotagmin-1, serine/ariginine-rich splicing factor 2, heat shock 70 kDa, ribonucleic particle-specific U1A and ribosomal protein S13. In addition, we found that several other proteins are able to bind to their own mRNAs. A large-scale analysis of biological pathways revealed that aggregation-prone and structurally disordered proteins have the highest propensity to interact with cognate RNAs. These findings are substantiated by experimental evidence on amyloidogenic proteins such as TAR DNA-binding protein 43 and fragile X mental retardation protein. Among the amyloidogenic proteins, we predicted that Parkinson’s disease-related α-synuclein is highly prone to interact with cognate transcripts, which suggests the existence of RNA-dependent factors in its function and dysfunction. Indeed, as aggregation is intrinsically concentration dependent, it is possible that autogenous interactions play a crucial role in controlling protein homeostasis.

## INTRODUCTION

Although proteins are involved in almost every cellular process, increasing evidence indicates that coding and non-coding RNAs play fundamental roles in gene regulation (1,2) and disease (3,4). Recent studies showed that establishment of aberrant associations or disruption of functional protein–RNA interactions occurs in neurological disorders (5,6). For instance, interaction with RNA favors conversion of alpha-helix rich prion protein PrP^C^ into the pathogenic beta-structure-rich insoluble conformer PrP^Sc^ that propagates in Creutzfeldt–Jakob disease (7). In Alzheimer’s disease, the association between Amyloid Precursor Protein mRNA and iron regulatory protein 1 is disrupted, resulting in compromised translation efﬁciency and elevated cytotoxicity (8).

Protein–RNA associations regulate several processes such as synthesis, folding, translocation, assembly and clearance of molecules. Previous studies suggested that ribonucleoprotein interactions might be able to facilitate protein and RNA folding (9,10). As a matter of fact, it has been observed that there is strong affinity between amino acids and their corresponding codons (11,12), which could imply a direct interaction between proteins and their own mRNAs (13,14). Indeed, TAR DNA-binding protein 43 (TDP-43) and Fragile X Mental Retardation protein (FMRP) have been found to interact with their own mRNAs (15,16). In these cases, expression is regulated by a negative feedback loop involving the 3′ untranslated region (UTR). Other autogenous associations have been observed in proteins associated with cell proliferation and gene expression (17,18). Also structurally disordered proteins such as Serine/Arginine-rich splicing factor 2 (SRSF2) (19) as well as heterogeneous ribonucleoprotein members (20,21) are able to inhibit their translation by associating with their own mRNAs.

How often do autogenous associations occur in the human proteome? Recent technological advances revealed that a large number of proteins have RNA-binding abilities (22), which suggests that interaction with cognate mRNAs could be more frequent than previously thought. Are autogenous associations linked to specific functions? It is possible that autoregulatory mechanisms are involved in controlling protein production. For instance, in the case of TDP-43 and FMRP, inhibition of expression via autogenous interaction is a way to preserve protein functionality (15,16). Overexpression leads to high protein production and enhanced amyloidogenicity, resulting in harmful gain- or loss-of-function effects on cellular metabolism (23).

In this work, we focused on the ability of proteins to establish autogenous associations. Using our computational approach *cat*RAPID (24), we studied the occurrence of these interactions in the human proteome. A large-scale analysis was performed to identify the role of autogenous associations in biological pathways and characterize their properties.

## MATERIALS AND METHODS

### Biological pathway annotations

We downloaded (September 2012) pathway data from two manually curated and high-quality resources: Reactome (25) and the NCI Pathway Interaction Database (NCI-PID) (26). The Reactome annotations (version 41) were gathered via the BioMart query interface returning a list of 167 canonical pathways containing 5375 unique protein coding genes, whereas the NCI-PID pathways were fetched directly from the database website (241 pathways, 2053 unique protein coding genes). In both cases, UniprotKB (27) accession numbers were converted to Ensembl (version 68) gene identifiers using the UniprotKB id-mapping file (version 2012_08). Subsequently, the gene pathway annotations were transferred to the corresponding polypeptides and coding/non-coding transcripts.

### Protein–RNA interaction prediction

We used the *cat*RAPID algorithm (24) to predict interaction propensity among all peptides and transcripts belonging to Reactome and NCI-PID pathways. *cat*RAPID was trained on a large set of protein–RNA pairs available in the Protein Data Bank to discriminate interacting and non-interacting molecules using secondary structure propensities, hydrogen bonding and van der Waals contributions (28). The method was tested on the non-nucleic acid-binding database (NNBP; area under the receiver operating characteristic curve of 0.92), the NPInter database (area under the receiver operating characteristic curve of 0.88) and a number of individual interactions (e.g. RNAse mitochondrial RNA MRP and X-inactive specific transcript XIST networks; average accuracy of 78%). Owing to CPU limitations in the calculation (29), we restricted the predictions to RNA sequences with a length between 50 and 1500 nt as well as to polypeptides with a length between 50 and 750 amino acids. The ‘fragment’ and ‘strength’ algorithms were used to identify regions involved in the binding and compute the specificity with respect to random protein–RNA associations (5,29). For each protein–RNA pair under investigation, a reference set of 10^2^ protein and 10^2^ RNA molecules (total of 10^4^ interactions) was used as a control (29). Reference sequences have the same lengths as the pair of interest to guarantee that the measure is independent of protein and RNA lengths (29).

### Gene partition

The gene partition function 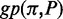 depends on the interaction propensity 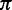 and type of protein–RNA association, which is defined as autogenous (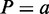), intra- (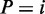) or inter-pathway (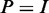):
(1)
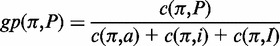



The number of counts 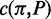 is the fraction 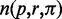 of protein (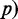 and RNA (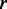) molecules with interaction propensity higher than 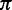:
(2)
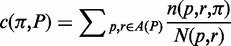



The function 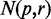 is the total number of interactions in the autogenous, intra- or inter-pathways 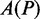


### Disorder propensity

We predicted disorder propensities using the IUPred algorithm (30) with the ‘long disorder’ prediction option. We defined a residue as disorder prone if its IUPred score was above 0.5, as in a previous experimental study (31).

### Comparison of the propensity distributions

We analyzed the disorder propensity of proteins involved in autogenous interactions by comparing them with the distributions of all the proteins annotated in the respective pathway data set. We evaluated the statistical significance using the Kolmogorov–Smirnov test (two-sided, alpha = 0.05).

### Pathway enrichment analysis

We assessed the enrichment of autogenous interactions in biological pathways using the Gene Set Enrichment Analysis (GSEA) method (32). For each pathway data set, we used as background the whole list of autogenous interactions predicted by the *cat*RAPID algorithm. We tested only those pathways annotated with autogenous interactions and containing at least five and not >500 genes (Supplementary Tables S1–4). We ran GSEA with default parameters and performing 1000 permutations.

### Protein and RNA abundances

Protein abundances were retrieved from the integrated whole organism Human PeptideAtlas (33,34), as assembled in http://pax-db.org (versions 2009, 2010, 2011 and 2012). Spectral counting from mass spectrometry was normalized to overall abundance and expressed as p.p.m. (parts per million) (35). RNA levels were taken from Gencode version 7 (36,37) averaging non-zero abundances in all available tissues. Transcript abundance, expressed in p.p.m., was estimated from RNA-seq experiments by normalizing the counts to total number of reads (36,38). For the calculation of protein–RNA interactions, 2487 proteins were combined with their RNAs (8976 transcripts considering the isoforms).

## RESULTS

### Protein–RNA interactions in biological pathways

Using the *cat*RAPID method (5,29), we systematically investigated the role of autogenous interactions in biological networks. For this purpose, we collected protein and RNA sequences annotated in two pathway resources: Reactome (25) and NCI-PID (26).

**Figure 1.**
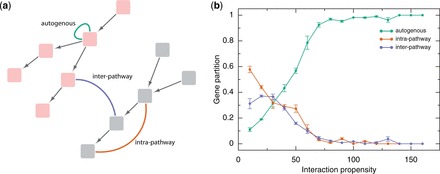
Autogenous versus intra- and inter-pathways interactions*.* (**a**) Sketch of biological pathways (pink and gray boxes connected by arrows; Supplementary Table S1). For each pathway, we studied three types of protein–RNA interactions: autogenous (green line), intra- (orange line) and inter-pathway (blue line); (**b**) From low to high interaction propensities (24), we found that the autogenous associations dominate over intra- and inter-pathway interactions present in Reactome (statistics for coding genes is shown; mean and s.e.m. of bins are shown; Supplementary Figure S1) (25). The gene partition is defined as the total fraction of genes showing preferential enrichment for autogenous, intra- or inter-pathway interactions (propensity >50: number n of genes enriched in autogenous interactions = 1238 of 1704; propensity > 100: *n* = 211 of 242; propensity >150: *n* = 20 of 20; Supplementary Figure S2; ‘Materials and Methods’ section).

We first computed the interaction potential of 295 × 10^6^ protein–RNA pairs (10 376 protein sequences against 28 493 RNA sequences) in Reactome and 65 × 10^6^ protein–RNA pairs (4754 protein sequences against 13 608 RNA sequences) in NCI-PID (Supplementary Table S1; ‘Materials and Methods’ section). We then classified interactions as follows (Figure 1a): (i) intra-pathway or between proteins and RNAs coded by different genes belonging to the same pathway; (ii) inter-pathways or between proteins and RNAs coded by different genes belonging to different non-overlapping pathways; and (iii) autogenous or between proteins and RNAs coded by the same genes. To quantify the proportion of genes that are preferentially involved in intra-, inter-pathways or autogenous interactions, we introduced the ‘gene partition’ function, which is the fraction of associations predicted at a certain interaction score (Figure 1b; ‘Materials and Methods’ section).

In both Reactome and NCI-PID (Figure 1b; Supplementary Figure S1), we found that intra-pathway and inter-pathway interactions are strongly depleted at high interaction propensities, whereas autogenous associations are enriched (Figure 1b; ‘Materials and Methods’ section). We observed the same trend for both coding and non-coding transcripts (Supplementary Figure S1).

### Biological pathways enriched in autogenous interactions

To uncover biological processes in which autogenous interactions play a functional role, we performed a GSEA (32) on both Reactome and NCI-PID pathways. In Reactome, we found 10 pathways enriched and 4 pathways depleted in autogenous interactions with a false discovery rate <5% (Supplementary Table S2). The top enriched pathway is ‘Amyloids’ (q-value = 3.2 × 10^−^^3^) followed by ‘Base Excision Repair’ (q-value = 3.8 × 10^−^^3^) and ‘Amine compound SLC transporters’ (q-value = 4.4 × 10^−^^3^) (Supplementary Table S2). Similarly, we identified 13 NCI-PID enriched pathways in autogenous interactions (Supplementary Table S3). We did not find any significantly depleted pathway at false discovery rate <5%. The top enriched NCI-PID pathways are ‘Signaling events mediated by HDAC Class III’ (q-value = 2.9 × 10^−^^2^), ‘C-MYC pathway’ (q-value = 3.0 × 10^−^^2^) and ‘Hypoxic and oxygen homeostasis regulation of HIF-1-alpha’ (q-value = 3.1 × 10^−^^2^) (Supplementary Table S3). We identified the ‘Botulinum neurotoxicity’/’Effect of Botulinum toxin’ pathway enriched in both databases (Reactome, q-value = 1.1 × 10^−^^2^; NCI-PID, q-value= 3.5 × 10^−^^2^) as well as the ‘α-synuclein signaling’ pathway enriched in NCI-PID (q-value = 4.2 × 10^−^^2^) and related to the Reactome ‘Amyloids’ pathway (Supplementary Table S3).

### Known autogenous interactions in biological pathways

To assess whether known cases of autogenous interactions were linked to the pathways identified in our analysis, we carried out a literature search. Indeed, amyloidogenic proteins such as TDP-43 and FMRP (Reactome pathway ‘Amyloids’) have strong propensities to bind to their mRNAs and have been discussed in our previous work (5).

**Figure 2.**
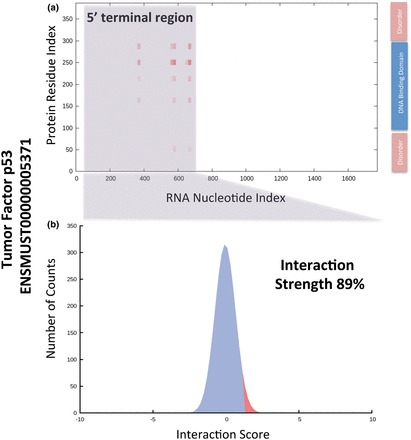
The autogenous interaction of tumor suppressor p53. (**a**) Using the *cat*RAPID algorithm (5,29), we were able to reproduce experimental evidence on the autogenous interaction of tumor suppressor p53 (17). The binding site is located in the 5′ terminal region of the mRNA (the gray box marks the region observed experimentally) (17). The interaction specificity between p53 and the 5′ terminal region of its mRNA was predicted to be significantly high (89%) with respect to a control set of protein–RNA associations (‘Materials and Methods’ section). The DNA binding domain and the disordered regions are reported as indicated in a recent study (41).

We found that tumor suppressor p53, involved in the two top-enriched pathways ‘Signaling events mediated by HDAC Class III’ and ‘Hypoxic and oxygen homeostasis regulation of HIF-1-alpha’ (both in NCI-PID), is able to bind to its own mRNA (17,39,40). In *Mus musculus*, the RNA-binding site of p53 is a stable stem-loop structure that involves the 5′ UTR plus a region of 280 nucleotides in the coding sequence (5′ terminal region) (17). A similar mechanism has been observed in *Homo Sapiens*, but no conclusive evidence has been reported on the interaction (39). In agreement with experimental evidence on murine p53, our predictions indicated that the 5′ terminal region has strong propensity to bind (Figure 2a), and the interaction is specific (interaction strength = 89%) with respect to a control set of molecules of same size (Figure 2b; ‘Materials and Methods’ section) (17). We found similar results for human p53, although the region involved in the binding is not known (Supplementary Figure S2a and b). It is worth mentioning that p53 has strong propensity to form amyloid fibrils (42), and interaction with nucleic acids represents a way to control its aggregation potential by limiting the amount of protein product (17,43). Moreover, we note that p53 can be associated with ‘Base Excision Repair’ pathway (Reactome) (44,45). Indeed, as ‘Base Excision Repair’ deficiency affects genome stability and is implicated in many human diseases, including premature aging neurodegeneration and cancer (46,47), we expect that a self-regulatory mechanism in its components could greatly contribute to system’s efficiency.

In both NCI-PID and Reactome, we found that the pathways ‘Botulinum neurotoxicity’ and ‘Effect of Botulinum toxin’ are significantly enriched in autogenous interactions. One of the key-players in Botulinum toxicity is synaptotagmin-1, which is essential in Ca(2+)-dependent neurotransmitter release (48). Synaptotagmin-1 interacts with the 3′ UTR of its own mRNA (49). In agreement with *in vitro* experiments, our predictions indicated that the 3′ UTR of synaptotagmin-1 RNA is involved in the autogenous interaction (Supplementary Figure S3a and b). Importantly, translation of synaptotagmin-1 is downregulated by the 3′ UTR, which is compatible with a negative feedback mechanism (49). As reported by light-scattering assays and electron microscopy, the protein forms large aggregates in a calcium-dependent manner (48), suggesting that the autogenous interaction protects against production of toxic oligomers (49).

Thymidylate synthase catalyzes the reaction generating thymidine monophosphate, which is phosphorylated to thymidine triphosphate for use in DNA synthesis and repair. Thymidylate synthase forms a ribonucleoprotein complex with C-MYC mRNA (50) and interacts with its own mRNA (51). The protein is not reported in the ‘C-MYC pathway’ (NCI-PID), but solid evidence exists on its interaction with C-MYC network (52). The RNA binding site for thymidylate synthase is within the 5′ UTR of the transcript (52). *cat*RAPID correctly located the interaction within the first 188 nt of the 5′ UTR (Supplementary Figure S4a) and predicted high specificity for the binding (interaction strength = 99%; Supplementary Figure S4b). We note that the bacterial homologue of thymidylate synthase is able to associate with its cognate mRNA (53), which highlights the crucial role of autogenous interactions across different species.

### Autogenous interactions and structural disorder

**Figure 3.**
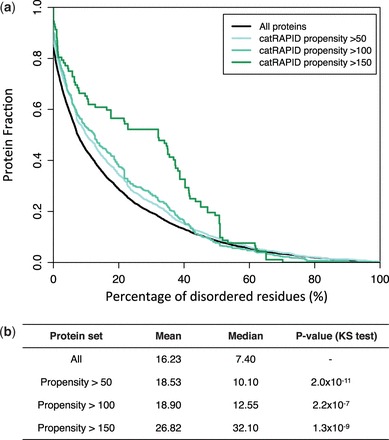
Structural disorder of proteins involved in autogenous interactions. (**a**) From low to high protein–RNA interaction propensities (24), we observed an increase in percentage of disorder residues (30), which is in agreement with previous experimental evidence (31) (Supplementary Figure S5). (**b**) The average and median values for the percentage of disordered residues are reported at different interaction propensities (Reactome database; propensity >50: number n of proteins = 1521; propensity >100: *n* = 353; propensity >150: *n* = 57). The statistical significance was assessed with the Kolmogorov–Smirnov test (KS test).

A recent study (31) showed that many RNA-binding proteins contain intrinsically disordered regions. Using the IUPred algorithm (30), we investigated the role of structural disorder in biological pathways. We found that proteins involved in autogenous interactions have a significant higher fraction of disorder prone residues compared with all proteins annotated in Reactome and NCI-PID (Figure 3 and Supplementary Figure S5; same results were observed for both coding and non-coding RNAs). To investigate whether known cases of autogenous interactions are enriched in unstructured regions, we performed a literature search.

**Figure 4.**
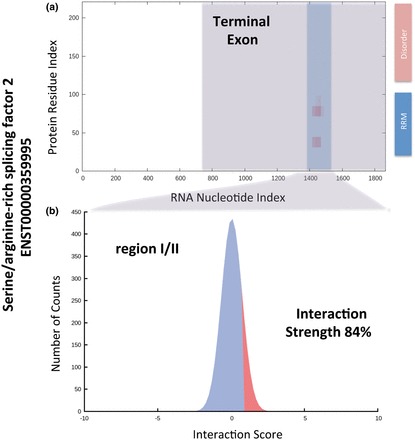
The autogenous interaction of SRSF2. (**a**) The catRAPID algorithm (5,29) was used to reproduce experimental evidence on the autogenous interaction of SRSF2 (19). The binding site is located in the final exon of SRSF2 mRNA (the blue box marks the region I/II that was determined experimentally) (19). (**b**) The interaction strength between SRSF2 and region I/II is significantly specific (84%) with respect to a control set of protein–RNA associations (‘Materials and Methods’ section). The RNA-binding domain RRM and disorder regions were reported as indicated in a previous study (54) and in agreement with the Uniprot (27) annotation (entry Q01130).

We found that the human SRSF2, which has a long disordered C-terminus spanning amino acids 117–221, interacts with its own transcript (19) (Figure 4a). Notably, *cat*RAPID correctly identified the binding site between the RRM domain (amino acids 14–92) and region I/II of the terminal exon (55) (Figure 4b; interaction strength = 84%). The disordered region was predicted to be not interacting with the terminal exon. As a matter of fact, the C-terminal region participates in processes that only indirectly relate to the RNA-binding activity of the protein: facilitation of Ser/Arg phosphorylation to allow entrance in the nucleus (56) and establishment of low-affinity interactions to enhance the splicing activity (57).

Human Heat Shock 70 kDa (HSP70) interacts with its own mRNA (58) and has a disordered C-terminal region of ∼10 kDa [highly conserved across species (59)] containing the Glu-Glu-Val-Asp regulatory motif. Using *cat*RAPID, we predicted that the binding occurs at the 3′ UTR, which is in agreement with previous observations (Supplementary Figure S6a) (60). Indeed, HSP70 has a strong tendency to bind to AU-rich sequences that are located at the 3′ UTR (Supplementary Figure S6b) (61). Importantly, we predicted that both the N-terminal ATPase domain and the disordered C-terminus are involved in the interaction, which is consistent with the observation that HSP70 RNA-binding affinity depends on the ATPase domain but is considerably reduced when the disordered region is removed (62) (Supplementary Figure S6c). A high concentration of HSP70 is toxic, as the protein has a strong tendency to aggregate (58). Hence, interaction with mRNA represents a mechanism to control protein production and formation of toxic aggregates.

The small nuclear RiboNucleic Particle-specific U1A protein also binds to its own transcript with high affinity and specificity (18). Human U1A protein comprises two RNP domains separated by a disordered linker containing a nuclear localization signal. The determinants of protein–RNA specificity reside within amino acids 1–102 of the first RNP domain and a region in the linker (63). Using *cat*RAPID, we predicted interactions between the 3′ UTR and the first RNP domain as well as a region of the disordered linker (Supplementary Figure S7a and b; protein domains take from UniProtKB entry P09012) (18). We also predicted interaction within the second RNP domain, which has not been reported in literature but is involved in pre-mRNAs recognition (64). We note that unstructured regions of the protein participate in base recognition by forming direct and water-mediated hydrogen bonds with RNA (65).

Also ribosomal protein S13 shows moderate presence of secondary structure (66) and is able to bind to its own mRNA (67). Indeed, even though no information is available on its native state, it is possible that S13 contains disordered regions as most ribosomal proteins (68,69). In agreement with experimental evidence, *cat*RAPID identified S13 binding site within the first and second exon (Supplementary Figure S8a and b) (67).

We note that also p53 and synaptotagmin-1 contain disordered regions, as shown by previous studies (41,70). In the calculations of autogenous interactions involving disordered proteins, we used protein and RNA sequences as reported in the original papers. As the length of the RNA sequences exceeded *cat*RAPID size limitation, the ‘fragmentation’ algorithm (5,29) was used to identify regions involved in the binding (‘Materials and Methods’ section).

### Autogenous interactions in control of protein translation

Autogenous interactions regulate gene expression at the translational level by controlling protein concentration. If protein concentration is high, binding to mRNA is expected to have a major effect on translation efficiency.

**Figure 5.**
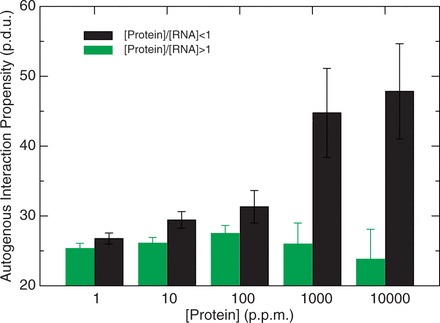
Autogenous interactions in control of protein translation. From low to high expression levels, increase in autogenous interaction propensity (black bars) is associated with reduced translation efficiency [(protein)/(RNA) < 1], which is compatible with a negative feedback loop mechanism. When the propensity for autogenous interaction is low (green bars), translation efficiency is high [(protein)/(RNA) >1], suggesting that the feedback loop mechanism is not active. Protein and RNA abundances were taken from PeptideAtlas (33,34) and GenCode version 7 (37) (error bars are standard deviations on interaction propensities; p.d.u. is procedure defined unit; p.p.m. is parts per million).

We investigated the relationship between protein abundance and propensity for autogenous interactions. From low to high expression levels, we found that reduction in translation efficiency (protein to RNA abundance ratio <1) is accompanied with an increase in autogenous interaction propensity (Figure 5), which is fully compatible with a negative feedback loop mechanism. If the negative feedback loop is active, translation slows down, and the ratio between protein and RNA abundance decreases. By contrast, in case of efficient translation (protein to RNA abundance ratio >1), the propensity for autogenous interactions is low (Figure 5), suggesting that the feedback loop does not occur.

Hence, our findings indicate that autogenous interactions might be involved in the modulation of protein expression by inducing regulatory feedback loops at the translational level. According to our analysis and in agreement with previous reports (15,49,58), negative feedback loops are a common type of mechanism involving autogenous interactions (for a list of predictions, see Supplementary Table S4).

### A hypothesis on alpha-synuclein association

Among the amyloid proteins, we predicted that α-synuclein (*SNCA* gene) is significantly prone to autogenous interactions. The average interaction propensity with *SNCA* transcripts = 68 ± 4, and *P* = 1.2 × 10^−^^12^ (the average interaction propensity with transcripts coding for the major protein isoform = 72 ± 5 and *P* = 1.3 × 10^−^^8^; *P*-values were calculated with Mann–Whitney U test). The most abundant isoforms ENST00000394986, ENST00000336904 and ENST00000394991 (36,37) have interaction propensities of 81, 71 and 61, respectively. Intriguingly, the transcripts coding for the major protein isoform have a protein to RNA abundance ratio of 0.58, which is compatible with a negative feedback mechanism. The protein has been found to be present in both the cytoplasm and nucleus (71,72),

Alpha-synuclein is a 14 kDa protein composed of an amphipathic, positively charged 100 residue N-terminal domain with a lysine-rich N-terminus that binds reversibly to anionic membranes (73) and a 40-residue acidic C-terminal domain (74). The protein is predominantly monomeric in solution with a smaller fraction of multimeric species and is intrinsically unstructured (75,76). Importantly, interactions with double- or single-stranded DNA are able to convert α-synuclein into a highly structured protein (77). Circular dichroism shows that the α-helical content increases from 5 to 64% upon binding to DNA, whereas the random coil decreases from 95 to 33% (77).

**Figure 6.**
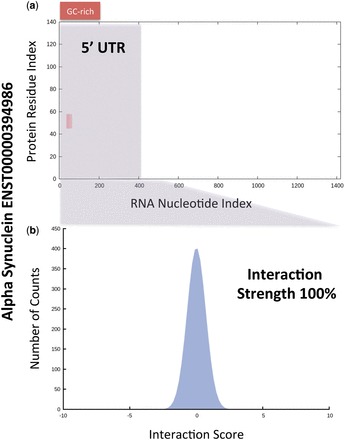
A hypothesis on the autogenous association of α-synuclein. Among the amyloid proteins, we found that α-synuclein (*SNCA* gene) is significantly prone to autogenous interactions (average interaction propensity with *SNCA* transcripts = 68 ± 4). (**a**) Our calculations indicated that transcript ENST00000394986, which is abundant in brain, binds to α-synuclein at the 5′ UTR (gray box). (**b**) The 5′ UTR association was predicted to be specific (interaction strength = 100%) and involving a GC-rich region (Supplementary Figure S9), in agreement with previous experimental evidence showing that α-synuclein binds to G and C nucleotides (77).

The fact that α-synuclein interacts with DNA suggests that nucleic acid interactions might be relevant for its regulation. Our calculations indicate that ENST00000394986, ENST00000336904 and ENST00000394991 bind to α-synuclein at the 5′ UTRs (Figure 6a and Supplementary Figure S9). We predicted that the 5′ UTR interaction is specific (the interaction strengths of ENST00000394986, ENST00000336904 and ENST00000394991 are 100, 95 and 99%, respectively; see Figure 6b) and within GC-rich regions (Supplementary Figure S10), in agreement with previous evidence showing α-synuclein preference for G and C nucleotides (77). Moreover, a lysine-rich region spanning residues 40–60 was predicted by *cat*RAPID to be involved in RNA recognition, which is consistent with previous results indicating an anion binding ability of the N-terminus (72).

At present, it is unknown whether RNA associations protect α-synuclein against formation of toxic aggregates. As a matter of fact, interaction with DNA sensibly increases α-synuclein amyloidogenicity (78) and a study on the Hofmeister series showed that anion binding promotes fibrillization (79). As GC-rich DNA aptamers have been found to associate with both monomeric and oligomeric α-synuclein (80), it is likely that stable RNA secondary structures, enriched in GC content, facilitate the disorder-to-order transition of α-synuclein, which could result in production of partially folded and highly amyloidogenic intermediates.

## DISCUSSION

In this work, we used the *cat*RAPID method (24) to compute the propensity of proteins to interact with coding and non-coding transcripts. In a number of biological pathways annotated in Reactome and NCI-PID, we found enrichment for autogenous interactions.

Our results are in agreement with available experimental evidence on the amyloidogenic TDP-43 and FMRP (the ‘Amyloids’ pathway has the highest enrichment in autogenous interactions) (15,16), tumor suppressor p53 (‘Signaling events mediated by HDAC Class III’, ‘Hypoxic and oxygen homeostasis regulation of HIF-1-alpha’ and ‘Base Excision Repair’) (39), synaptotagmin-1 (‘Botulinum neurotoxicity’ and ‘Effect of Botulinum toxin’) (49) and thymidylate synthase (connected to ‘C-MYC pathway’) (51). We expect that other autogenous interactions will occur in these pathways, although few cases have been reported in literature. For instance, the RNA-binding chaperone HSP90 (81,82) is involved in ‘Hypoxic and oxygen homeostasis regulation of HIF-1-alpha’ pathway, and it might have the ability to associate with its own transcript as other heat shock proteins (58,83). Moreover, we found enrichment in pathways such as ‘amine compound SLC transporters’, ‘metabolism of nitric oxide’, ‘iron uptake and transport’, ‘ABC-family proteins mediated transport’, ‘metabolism of vitamins and cofactors’, ‘amino acid transport’, ‘energy metabolism’, where autogenous interactions could be playing a role in metabolic regulation (84).

Our analysis showed that disordered proteins have significant propensity for autogenous interactions. The results are in agreement with experimental evidence on SRSF2 (19), HSP70 (58), U1A (18), p53 (17) and synaptotagmin-1 (49). The fact that proteins containing disordered regions have a high potential for autogenous interactions suggests that RNA interactions could protect unstructured domains from aberrant interactions or aggregation (85,86). Indeed, it has been observed that polyanionic molecules increase the solubility of nascent polypeptides, and that RNA molecules can act as molecular chaperones helping proteins to fold into their native structures (87). As a matter of fact, interaction with cognate nucleic acids directly influences the aggregation propensity of TDP-43 (15,88), FMRP (89,90), p53 (17,43), synaptotagmin-1 (48,49) and HSP70 (58,83).

In the ‘Amyloids’ pathway, we focused on α-synuclein, a highly disordered and amyloidogenic protein linked to pathogenetic processes such as Parkinson’s disease and Lewy body dementia. Previous evidence indicated that α-synuclein forms partially folded multimers and aggregates impairing neuronal viability (75). We predicted that α-synuclein is able to establish autogenous interactions, which suggests the existence of RNA-dependent factors in the etiopathogenesis of Parkinson’s disease and other synucleinopathies. Indeed, our findings indicate that α-synuclein solubility might be modulated *in vivo* by associations with molecules such as nucleic acids (91,92). At present, it is has been shown that interaction with GC-rich DNA increases α-synuclein amyloidogenicity (77).

Although α-synuclein is present at the presynaptic terminals of neurons (93), several studies show that it can localize in the ‘nucleus’ (94,95). It should be considered that some mRNAs are shuttled to the axonal periphery where local synthesis takes place (96). Hence, the α-synuclein localization pattern and the presence of mRNAs at the neuronal terminals suggest that the predicted interaction of α-synuclein with RNA molecules may occur in the cellular context.

As in the case of other ribonucleoprotein interactions (28,97), we expect that ancillary elements present *in vivo* could increase α-synuclein affinity for nucleic acid associations. We do not exclude that the ribosomal components themselves could contribute to the formation of autogenous associations (10,98). As a matter of fact, the ribosome is the cellular component where autogenous interactions and translational control could take place simultaneously. Although our predictions on α-synuclein are compatible with a feedback loop mechanism (15,16), experimental evidence is required to determine the binding affinity of α-synuclein for RNA molecules and evaluate whether the interactions are mediated by other factors present in the cellular context.

Our findings suggest that autogenous interactions are able to reduce protein expression by inducing a negative feedback loop at the translational level. We previously observed that a tight anti-correlation (97%) exists between mRNA expression levels and aggregation rates of proteins (99,100). This relationship suggests that an evolutionary pressure acts against formation of toxic aggregates (101), and a molecular mechanism is in place to control expression of amyloidogenic proteins (102). In the light of our new findings, it is possible to speculate that autogenous interactions directly reduce the aggregation potential of proteins by controlling expression via feedback loops. As a number of genes have been reported to be dosage-sensitive (86,103), it is tempting to hypothesize that autogenous interactions play an important role in regulating their expression.

## SUPPLEMENTARY DATA

Supplementary Data are available at NAR Online.

## FUNDING

Ministerio de Economia y Competividad [SAF2011-26211 to G.G.T.], the European Research Council [ERC Starting Grant to G.G.T.] and the RTTIC project (to A.Z.); Programa de Ayudas FPI del Ministerio de Economia y Competitividad [BES-2012-052457 to D.M.]; and Marie Curie Fellowship [FP7 Cofound Action to B.B.]. Funding for open access charge: Ministerio de Economia y Competividad [SAF2011-26211 to G.G.T.] and the European Research Council [ERC Starting Grant to G.G.T.].

*Conflict of interest statement*. None declared.

## Supplementary Material

Supplementary Data

## References

[1] Guttman M, Rinn JL (2012). Modular regulatory principles of large non-coding RNAs. Nature.

[2] Lai F, Orom UA, Cesaroni M, Beringer M, Taatjes DJ, Blobel GA, Shiekhattar R (2013). Activating RNAs associate with Mediator to enhance chromatin architecture and transcription. Nature.

[3] Cooper TA, Wan L, Dreyfuss G (2009). RNA and disease. Cell.

[4] Johnson R, Noble W, Tartaglia GG, Buckley NJ (2012). Neurodegeneration as an RNA disorder. Prog. Neurobiol..

[5] Cirillo D, Agostini F, Klus P, Marchese D, Rodriguez S, Bolognesi B, Tartaglia GG (2013). Neurodegenerative diseases: quantitative predictions of protein-RNA interactions. RNA.

[6] Anthony K, Gallo J-M (2010). Aberrant RNA processing events in neurological disorders. Brain Res..

[7] Deleault NR, Lucassen RW, Supattapone S (2003). RNA molecules stimulate prion protein conversion. Nature.

[8] Cho H-H, Cahill CM, Vanderburg CR, Scherzer CR, Wang B, Huang X, Rogers JT (2010). Selective translational control of the Alzheimer amyloid precursor protein transcript by iron regulatory protein-1. J. Biol. Chem..

[9] Hogg JR, Collins K (2008). Structured non-coding RNAs and the RNP Renaissance. Curr. Opin. Chem. Biol..

[10] Herschlag D (1995). RNA chaperones and the RNA folding problem. J. Biol. Chem..

[11] Woese CR, Dugre DH, Saxinger WC, Dugre SA (1966). The molecular basis for the genetic code. Proc. Natl Acad. Sci. USA.

[12] Woese CR (1967). The Genetic Code: The Molecular Basis for Genetic Expression.

[13] Biro J, Benyo B, Sansom C, Szlavecz A, Fordos G, Micsik T, Benyo Z (2003). A common periodic table of codons and amino acids. Biochem. Biophys. Res. Commun..

[14] Hlevnjak M, Polyansky AA, Zagrovic B (2012). Sequence signatures of direct complementarity between mRNAs and cognate proteins on multiple levels. Nucleic Acids Res..

[15] Ayala YM, De Conti L, Avendaño-Vázquez SE, Dhir A, Romano M, D’Ambrogio A, Tollervey J, Ule J, Baralle M, Buratti E (2011). TDP-43 regulates its mRNA levels through a negative feedback loop. EMBO J..

[16] Schaeffer C, Bardoni B, Mandel J-L, Ehresmann B, Ehresmann C, Moine H (2001). The fragile X mental retardation protein binds specifically to its mRNA via a purine quartet motif. EMBO J..

[17] Mosner J, Mummenbrauer T, Bauer C, Sczakiel G, Grosse F, Deppert W (1995). Negative feedback regulation of wild-type p53 biosynthesis. EMBO J..

[18] Boelens WC, Jansen EJR, van Venrooij WJ, Stripecke R, Mattaj IW, Gunderson SI (1993). The human U1 snRNP-Specific U1A protein inhibits polyadenylation of its own pre-mRNA. Cell.

[19] Sureau A, Gattoni R, Dooghe Y, Stévenin J, Soret J (2001). SC35 autoregulates its expression by promoting splicing events that destabilize its mRNAs. EMBO J..

[20] Parakhnevitch NM, Ivanov AV, Malygin AA, Karpova GG (2007). Human ribosomal protein S13 inhibits splicing of its own pre-mRNA. Mol. Biol..

[21] Johnsen M, Christensen T, Dennis PP, Fiil NP (1982). Autogenous control: ribosomal protein L10-L12 complex binds to the leader sequence of its mRNA. EMBO J..

[22] Riley KJ, Steitz JA (2013). The ‘Observer Effect’ in genome-wide surveys of protein-RNA interactions. Mol. Cell.

[23] Budini M, Buratti E (2011). TDP-43 autoregulation: implications for disease. J. Mol. Neurosci..

[24] Bellucci M, Agostini F, Masin M, Tartaglia GG (2011). Predicting protein associations with long noncoding RNAs. Nat. Methods.

[25] Croft D, O’Kelly G, Wu G, Haw R, Gillespie M, Matthews L, Caudy M, Garapati P, Gopinath G, Jassal B (2011). Reactome: a database of reactions, pathways and biological processes. Nucleic Acids Res..

[26] Schaefer CF, Anthony K, Krupa S, Buchoff J, Day M, Hannay T, Buetow KH (2009). PID: the pathway interaction database. Nucleic Acids Res..

[27] UniProt Consortium (2012). Reorganizing the protein space at the Universal Protein Resource (UniProt). Nucleic Acids Res..

[28] Cirillo D, Agostini F, Tartaglia GG (2013). Predictions of protein–RNA interactions. Wiley Interdiscip. Rev. Comput. Mol. Sci..

[29] Agostini F, Cirillo D, Bolognesi B, Tartaglia GG (2013). X-inactivation: quantitative predictions of protein interactions in the Xist network. Nucleic Acids Res..

[30] Dosztányi Z, Csizmok V, Tompa P, Simon I (2005). IUPred: web server for the prediction of intrinsically unstructured regions of proteins based on estimated energy content. Bioinformatics.

[31] Castello A, Fischer B, Eichelbaum K, Horos R, Beckmann BM, Strein C, Davey NE, Humphreys DT, Preiss T, Steinmetz LM (2012). Insights into RNA biology from an atlas of mammalian mRNA-binding proteins. Cell.

[32] Subramanian A, Tamayo P, Mootha VK, Mukherjee S, Ebert BL, Gillette MA, Paulovich A, Pomeroy SL, Golub TR, Lander ES (2005). Gene set enrichment analysis: A knowledge-based approach for interpreting genome-wide expression profiles. Proc. Natl Acad. Sci. USA.

[33] Deutsch EW, Eng JK, Zhang H, King NL, Nesvizhskii AI, Lin B, Lee H, Yi EC, Ossola R, Aebersold R (2005). Human Plasma PeptideAtlas. Proteomics.

[34] Farrah T, Deutsch EW, Omenn GS, Campbell DS, Sun Z, Bletz JA, Mallick P, Katz JE, Malmström J, Ossola R (2011). A high-confidence human plasma proteome reference set with estimated concentrations in PeptideAtlas. Mol. Cell. Proteomics.

[35] Wang M, Weiss M, Simonovic M, Haertinger G, Schrimpf SP, Hengartner MO, Mering C, von Mering C (2012). PaxDb, a database of protein abundance averages across all three domains of life. Mol. Cell. Proteomics.

[36] Harrow J, Denoeud F, Frankish A, Reymond A, Chen C-K, Chrast J, Lagarde J, Gilbert JGR, Storey R, Swarbreck D (2006). GENCODE: producing a reference annotation for ENCODE. Genome Biol..

[37] Harrow J, Frankish A, Gonzalez JM, Tapanari E, Diekhans M, Kokocinski F, Aken BL, Barrell D, Zadissa A, Searle S (2012). GENCODE: the reference human genome annotation for The ENCODE Project. Genome Res..

[38] Roberts A, Trapnell C, Donaghey J, Rinn JL, Pachter L (2011). Improving RNA-Seq expression estimates by correcting for fragment bias. Genome Biol..

[39] Mokdad-Gargouri R, Belhadj K, Gargouri A (2001). Translational control of human p53 expression in yeast mediated by 5′-UTR–ORF structural interaction. Nucleic Acids Res..

[40] Lu X (2010). Tied up in loops: positive and negative autoregulation of p53. Cold Spring Harb. Perspect. Biol..

[41] Xue B, Brown CJ, Dunker AK, Uversky VN (2013). Intrinsically disordered regions of p53 family are highly diversified in evolution. Biochim. Biophys. Acta.

[42] Xu J, Reumers J, Couceiro JR, De Smet F, Gallardo R, Rudyak S, Cornelis A, Rozenski J, Zwolinska A, Marine J-C (2011). Gain of function of mutant p53 by coaggregation with multiple tumor suppressors. Nat. Chem. Biol..

[43] Ishimaru D, Ano Bom AP, Lima LM, Quesado PA, Oyama MF, de Moura Gallo CV, Cordeiro Y, Silva JL (2009). Cognate DNA stabilizes the tumor suppressor p53 and prevents misfolding and aggregation. Biochemistry.

[44] Zhou J, Ahn J, Wilson SH, Prives C (2001). A role for p53 in base excision repair. EMBO J..

[45] Zurer I, Hofseth LJ, Cohen Y, Xu-Welliver M, Hussain SP, Harris CC, Rotter V (2004). The role of p53 in base excision repair following genotoxic stress. Carcinogenesis.

[46] Lombard DB, Chua KF, Mostoslavsky R, Franco S, Gostissa M, Alt FW (2005). DNA repair, genome stability, and aging. Cell.

[47] Caldecott KW (2008). Single-strand break repair and genetic disease. Nat. Rev. Genet..

[48] Damer CK, Creutz CE (1996). Calcium-dependent self-association of synaptotagmin I. J. Neurochem..

[49] Sukumaran SS, Banerjee S, Bhasker S, Thekkuveettil A (2008). The cytoplasmic C2A domain of synaptotagmin shows sequence specific interaction with its own mRNA. Biochem. Biophys. Res. Commun..

[50] Mannava S, Moparthy KC, Wheeler LJ, Leonova KI, Wawrzyniak JA, Bianchi-Smiraglia A, Berman AE, Flanagan S, Shewach DS, Zeitouni NC (2012). Ribonucleotide reductase and thymidylate synthase or exogenous deoxyribonucleosides reduce DNA damage and senescence caused by C-MYC depletion. Aging (Albany NY).

[51] Chu E, Voeller D, Koeller DM, Drake JC, Takimoto CH, Maley GF, Maley F, Allegra CJ (1993). Identification of an RNA binding site for human thymidylate synthase. Proc. Natl Acad. Sci. USA..

[52] Chu E, Takechi T, Jones KL, Voeller DM, Copur SM, Maley GF, Maley F, Segal S, Allegra CJ (1995). Thymidylate synthase binds to c-myc RNA in human colon cancer cells and *in vitr*o. Mol. Cell. Biol..

[53] Voeller DM, Changchien LM, Maley GF, Maley F, Takechi T, Turner RE, Montfort WR, Allegra CJ, Chu E (1995). Characterization of a specific interaction between *Escherichia coli* thymidylate synthase and *Escherichia coli* thymidylate synthase mRNA. Nucleic Acids Res..

[54] Haynes C, Iakoucheva LM (2006). Serine/arginine-rich splicing factors belong to a class of intrinsically disordered proteins. Nucleic Acids Res..

[55] Dreumont N, Hardy S, Behm-Ansmant I, Kister L, Branlant C, Stevenin J, Bourgeois CF (2010). Antagonistic factors control the unproductive splicing of SC35 terminal intron. Nucleic Acids Res..

[56] Twyffels L, Gueydan C, Kruys V (2011). Shuttling SR proteins: more than splicing factors. FEBS J..

[57] Liu H-X, Chew SL, Cartegni L, Zhang MQ, Krainer AR (2000). Exonic splicing enhancer motif recognized by human SC35 under splicing conditions. Mol. Cell Biol..

[58] Balakrishnan K, De Maio A (2006). Heat shock protein 70 binds its own messenger ribonucleic acid as part of a gene expression self-limiting mechanism. Cell Stress Chaperones.

[59] Smock RG, Blackburn ME, Gierasch LM (2011). Conserved, disordered C terminus of DnaK enhances cellular survival upon stress and DnaK *in vitro* chaperone activity. J. Biol. Chem..

[60] Henics T (2003). Extending the ‘stressy’ edge: molecular chaperones flirting with RNA. Cell Biol. Int..

[61] Kishor A, Tandukar B, Ly YV, Toth EA, Suarez Y, Brewer G, Wilson GM (2013). Hsp70 is a novel posttranscriptional regulator of gene expression that binds and stabilizes selected mRNAs containing AU-rich elements. Mol. Cell. Biol..

[62] Zimmer C, von Gabain A, Henics T (2001). Analysis of sequence-specific binding of RNA to Hsp70 and its various homologs indicates the involvement of N- and C-terminal interactions. RNA.

[63] Allain FH, Howe PW, Neuhaus D, Varani G (1997). Structural basis of the RNA-binding specificity of human U1A protein. EMBO J..

[64] Lutz CS, Alwine JC (1994). Direct interaction of the U1 snRNP-A protein with the upstream efficiency element of the SV40 late polyadenylation signal. Genes Dev..

[65] Oubridge C, Ito N, Evans PR, Teo CH, Nagai K (1994). Crystal structure at 1.92 A resolution of the RNA-binding domain of the U1A spliceosomal protein complexed with an RNA hairpin. Nature.

[66] Malygin A, Parakhnevitch N, Karpova G (2005). Human ribosomal protein S13: cloning, expression, refolding, and structural stability. Biochim. Biophys. Acta.

[67] Parakhnevitch NM, Ivanov AV, Malygin AA, Karpova GG (2007). Human ribosomal protein S13 inhibits splicing of its own pre-mRNA. Mol. Biol..

[68] Tompa P, Csermely P (2004). The role of structural disorder in the function of RNA and protein chaperones. FASEB J..

[69] Korneta I, Bujnicki JM (2012). Intrinsic disorder in the human spliceosomal proteome. PLoS Comput. Biol..

[70] Sutton RB, Davletov BA, Berghuis AM, Sudhof TC, Sprang SR (1995). Structure of the first C2 domain of synaptotagmin I: a novel Ca2+/phospholipid-binding fold. Cell.

[71] Goers J, Manning-Bog AB, McCormack AL, Millett IS, Doniach S, Di Monte DA, Uversky VN, Fink AL (2003). Nuclear localization of alpha-synuclein and its interaction with histones. Biochemistry.

[72] Siddiqui A, Chinta SJ, Mallajosyula JK, Rajagopolan S, Hanson I, Rane A, Melov S, Andersen JK (2012). Selective binding of nuclear alpha-synuclein to the PGC1alpha promoter under conditions of oxidative stress may contribute to losses in mitochondrial function: implications for Parkinson’s disease. Free Radic. Biol. Med..

[73] Rhoades E, Ramlall TF, Webb WW, Eliezer D (2006). Quantification of alpha-synuclein binding to lipid vesicles using fluorescence correlation spectroscopy. Biophys. J..

[74] Burré J, Sharma M, Tsetsenis T, Buchman V, Etherton MR, Südhof TC (2010). Alpha-synuclein promotes SNARE-complex assembly *in viv*o and *in vitro*. Science.

[75] Burré J, Vivona S, Diao J, Sharma M, Brunger AT, Südhof TC (2013). Properties of native brain α-synuclein. Nature.

[76] Fauvet B, Mbefo MK, Fares M-B, Desobry C, Michael S, Ardah MT, Tsika E, Coune P, Prudent M, Lion N (2012). α-Synuclein in central nervous system and from erythrocytes, mammalian cells, and *Escherichia coli* exists predominantly as disordered monomer. J. Biol. Chem..

[77] Hegde ML, Rao KS (2007). DNA induces folding in α-synuclein: understanding the mechanism using chaperone property of osmolytes. Arch. Biochem. Biophys..

[78] Cherny D, Hoyer W, Subramaniam V, Jovin TM (2004). Double-stranded DNA stimulates the fibrillation of alpha-synuclein *in vitro* and is associated with the mature fibrils: an electron microscopy study. J. Mol. Biol..

[79] Munishkina LA, Henriques J, Uversky VN, Fink AL (2004). Role of protein-water interactions and electrostatics in alpha-synuclein fibril formation. Biochemistry.

[80] Tsukakoshi K, Abe K, Sode K, Ikebukuro K (2012). Selection of DNA aptamers that recognize α-synuclein oligomers using a competitive screening method. Anal. Chem..

[81] Huang YW, Hu CC, Liou MR, Chang BY, Tsai CH, Meng M, Lin NS, Hsu YH (2012). Hsp90 interacts specifically with viral RNA and differentially regulates replication initiation of bamboo mosaic virus and associated satellite RNA. PLoS Pathog..

[82] Castello A, Fischer B, Eichelbaum K, Horos R, Beckmann BM, Strein C, Davey NE, Humphreys DT, Preiss T, Steinmetz LM (2012). Insights into RNA biology from an atlas of mammalian mRNA-binding proteins. Cell.

[83] Henics T, Nagy E, Oh HJ, Csermely P, Gabain A, von Gabain A, Subjeck JR (1999). Mammalian Hsp70 and Hsp110 proteins bind to RNA motifs involved in mRNA Sstability. J. Biol. Chem..

[84] Hentze MW (1994). Enzymes as RNA-binding proteins: a role for (di)nucleotide-binding domains?. Trends Biochem. Sci..

[85] Choi SI, Ryu K, Seong BL (2009). RNA-mediated chaperone type for de novo protein folding. RNA Biol..

[86] Vavouri T, Semple JI, Garcia-Verdugo R, Lehner B (2009). Intrinsic protein disorder and interaction promiscuity are widely associated with dosage sensitivity. Cell.

[87] Frankel AD, Smith CA (1998). Induced folding in RNA-protein recognition: more than a simple molecular handshake. Cell.

[88] Huang Y-C, Lin K-F, He R-Y, Tu P-H, Koubek J, Hsu Y-C, Huang JJ-T (2013). Inhibition of TDP-43 aggregation by nucleic acid binding. PLoS One.

[89] Aschrafi A, Cunningham BA, Edelman GM, Vanderklish PW (2005). The fragile X mental retardation protein and group I metabotropic glutamate receptors regulate levels of mRNA granules in brain. Proc. Natl Acad. Sci. USA.

[90] Sjekloća L, Pauwels K, Pastore A (2011). On the aggregation properties of FMRP – a link with the FXTAS syndrome?. FEBS J.

[91] Devine MJ, Gwinn K, Singleton A, Hardy J (2011). Parkinson’s disease and α-synuclein expression. Mov. Disord..

[92] Martin I, Dawson VL, Dawson TM (2011). Recent advances in the genetics of Parkinson’s disease. Annu. Rev. Genomics Hum. Genet..

[93] Fortin DL, Troyer MD, Nakamura K, Kubo S, Anthony MD, Edwards RH (2004). Lipid rafts mediate the synaptic localization of alpha-synuclein. J. Neurosci..

[94] Yu S, Li X, Liu G, Han J, Zhang C, Li Y, Xu S, Liu C, Gao Y, Yang H (2007). Extensive nuclear localization of alpha-synuclein in normal rat brain neurons revealed by a novel monoclonal antibody. Neuroscience.

[95] Maroteaux L, Campanelli JT, Scheller RH (1988). Synuclein: a neuron-specific protein localized to the nucleus and presynaptic nerve terminal. J. Neurosci..

[96] Liu-Yesucevitz L, Bassell GJ, Gitler AD, Hart AC, Klann E, Richter JD, Warren ST, Wolozin B (2011). Local RNA translation at the synapse and in disease. J. Neurosci..

[97] Guerrier-Takada C, Gardiner K, Marsh T, Pace N, Altman S (1983). The RNA moiety of ribonuclease P is the catalytic subunit of the enzyme. Cell.

[98] Kovacs D, Rakacs M, Agoston B, Lenkey K, Semrad K, Schroeder R, Tompa P (2009). Janus chaperones: assistance of both RNA- and protein-folding by ribosomal proteins. FEBS Lett..

[99] Tartaglia GG, Pechmann S, Dobson CM, Vendruscolo M (2007). Life on the edge: a link between gene expression levels and aggregation rates of human proteins. Trends Biochem. Sci..

[100] Baldwin AJ, Knowles TP, Tartaglia GG, Fitzpatrick AW, Devlin GL, Shammas SL, Waudby CA, Mossuto MF, Meehan S, Gras SL (2011). Metastability of native proteins and the phenomenon of amyloid formation. J. Am. Chem. Soc..

[101] Calloni G, Chen T, Schermann SM, Chang H, Genevaux P, Agostini F, Tartaglia GG, Hayer-Hartl M, Hartl FU (2012). DnaK functions as a central hub in the *E. coli* chaperone network. Cell Rep..

[102] Gsponer J, Babu MM (2012). Cellular strategies for regulating functional and nonfunctional protein aggregation. Cell Rep..

[103] Moriya H, Makanae K, Watanabe K, Chino A, Shimizu-Yoshida Y (2012). Robustness analysis of cellular systems using the genetic tug-of-war method. Mol. Biosyst..

