# Supplementary material for: Principles of self-organization in biological pathways: a hypothesis on the autogenous association of alpha-synuclein
Source: Nucleic Acids Res. 2013 Sep 3;41(22):9987–98. doi: 10.1093/nar/gkt794 (PMC3905859; doi:10.1093/nar/gkt794)
Supplement: Supplementary Data [file supp_41_22_9987__index.html]

Principles of self-organization in biological pathways: a hypothesis on the autogenous association of alpha-synuclein — Principles of self-organization in biological pathways: a hypothesis on the autogenous association of alpha-synuclein — Supplementary Data 

# Principles of self-organization in biological pathways: a hypothesis on the autogenous association of alpha-synuclein

## Supplementary Data

files

**Files in this Data Supplement:**

- Supplementary Data - pdf file
