# Supplementary material for: Principles of self-organization in biological pathways: a hypothesis on the autogenous association of alpha-synuclein
Source: Nucleic Acids Res. 2013 Sep 3;41(22):9987–98. doi: 10.1093/nar/gkt794 (PMC3905859; doi:10.1093/nar/gkt794)
Supplement: Supplementary Data [file supp_gkt794_nar-01976-n-2013-File008.pdf]

Supplementary Information of

Principles of Self-Organization in Biological Pathways: A Hypothesis on the Autogenous Association of Alpha-Synuclein

Andreas Zanzoni, Domenica Marchese, Federico Agostini, Benedetta Bolognesi, Davide Cirillo, Maria Teresa Botta-Orfila, Carmen Maria Livi, Silvia Rodriguez-Mulero and Gian Gaetano Tartaglia

Supplementary Tables

	Pathways	Protein sequences	Coding RNA sequences	Non-coding RNA sequences	Non-coding RNA biotypes
**NCI-PID**	224	4754	5123	8485	
				3858	*Processed transcript*
				3556	*Retained intron*
				1071	*Non-sense meditated decay*
**Reactome**	167	10376	11304	17189	
				7946	*Processed transcript*
				6780	*Retained intron*
				2463	*Non-sense meditated decay*

Supplementary Table 1. *Number of protein and transcript sequences in the Reactome and NCI-PID pathways.* The statistics on coding and non-coding transcripts are reported separately. Non-coding RNA biotypes were collected from the Ensembl database.

Reactome pathway name	ES	NES	P-value	Q-value
Enriched				
**Amyloids**	**0.50**	**1.59**	**0**	**0.003230039**
Base Excision Repair	0.49	1.59	0	0.003798912
Amine compound SLC transporters	0.51	1.61	0	0.004434586
Metabolism of nitric oxide	0.52	1.61	0	0.006178905
Signal regulatory protein (SIRP) family interactions	0.51	1.63	0	0.010448562
Botulinum neurotoxicity	0.47	1.49	0	0.011695406
Elongation arrest and recovery	0.47	1.48	0	0.01314011
Latent infection of Homo sapiens with Mycobacterium tuberculosis	0.45	1.46	0	0.015884457
ABC-family proteins mediated transport	0.46	1.43	0.002	0.021284832
Iron uptake and transport	0.43	1.37	0	0.048071492
Transcriptional Regulation of White Adipocyte Differentiation	0.42	1.35	0.001	0.050647687
Metabolism of vitamins and cofactors	0.42	1.35	0	0.055165242
Collagen formation	0.42	1.34	0.001	0.060681045
Viral dsRNA:TLR3:TRIF Complex Activates TBK1/IKK epsilon	0.46	1.33	0.052525252	0.063186444
Bile salt and organic anion SLC transporters	0.43	1.31	0.023046093	0.07306761
Platelet homeostasis	0.39	1.28	0	0.10522428
APC-Cdc20 mediated degradation of Nek2A	0.41	1.28	0.008008008	0.10563387
Amino acid transport across the plasma membrane	0.39	1.27	0.01	0.110434674
Integration of energy metabolism	0.38	1.25	0	0.11530844
Potassium Channels	0.39	1.26	0.003	0.11546165
Signaling by BMP	0.41	1.25	0.039	0.11587791
Meiosis	0.38	1.24	0.001	0.117704734
Smooth Muscle Contraction	0.38	1.24	0.001	0.12061862
Aquaporin-mediated transport	0.39	1.26	0.001	0.12108914
Regulatory RNA pathways	0.40	1.24	0.056056056	0.12158073
Extracellular matrix organization	0.38	1.24	0.006	0.124391645
Meiotic Recombination	0.38	1.21	0.032	0.17208144
Metabolism of porphyrins	0.38	1.19	0.087	0.19959927
Depleted				
NICD traffics to nucleus	-0.27	-2.05	0	0
RORA Activates Circadian Expression	-0.32	-1.83	0	0.004366812
Regulation of beta-cell development	-0.24	-1.57	0	0.01746725
Organic anion transporters	-0.39	-1.40	0.05154639	0.024745269

Supplementary Table 2. *Gene set enrichment analysis of autogenous interactions in Reactome pathways.* For each pathway we report: enrichment score (ES), Normalized enrichment score (NES), the nominal p-value (P-value), and the adjusted P-value (Q-value). The table lists enriched and depleted pathways with a False Discovery Rate (FDR) < 20%.

NCI-PID pathway name	ES	NES	P-value	Q-value
Enriched				
Signaling events mediated by HDAC Class III	0.50	1.57	0	0.029423453
C-MYC pathway	0.51	1.62	0	0.030875338
Hypoxic and oxygen homeostasis regulation of HIF-1-alpha	0.47	1.50	0	0.031121688
Sphingosine 1-phosphate (S1P) pathway	0.51	1.48	0.006085193	0.03530931
Effects of Botulinum toxin	0.48	1.44	0.005005005	0.035695974
Arf6 trafficking events	0.45	1.45	0	0.03624375
PDGF receptor signaling network	0.50	1.46	0.009164969	0.03697721
Atypical NF-kappaB pathway	0.44	1.39	0	0.039326284
S1P5 pathway	0.52	1.51	0.003030303	0.039570443
E-cadherin signaling in the nascent adherens junction	0.44	1.40	0	0.039729886
Posttranslational regulation of adherens junction stability and dissassembly	0.43	1.39	0	0.040995207
**Alpha-synuclein signaling**	**0.44**	**1.41**	**0**	**0.04292989**
Calcium signaling in the CD4+ TCR pathway	0.44	1.41	0	0.047486212
EGF receptor (ErbB1) signaling pathway	0.42	1.36	0	0.057327013
S1P4 pathway	0.44	1.34	0.02	0.06289986
Syndecan-4-mediated signaling events	0.42	1.33	0.004	0.068733335
Regulation of cytoplasmic and nuclear SMAD2/3 signaling	0.43	1.32	0.007	0.07127475
Plasma membrane estrogen receptor signaling	0.42	1.31	0.001	0.078085385
Regulation of Ras family activation	0.40	1.28	0.002	0.08190428
Visual signal transduction: Cones	0.44	1.30	0.042424243	0.082054295
IGF1 pathway	0.40	1.30	0	0.08228388
Insulin-mediated glucose transport	0.40	1.28	0.005	0.084385656
ALK1 signaling events	0.41	1.30	0.004	0.08567474
Class IB PI3K non-lipid kinase events	0.54	1.28	0.1474501	0.08644842
IL2 signaling events mediated by PI3K	0.40	1.29	0.001	0.08652738
Neurotrophic factor-mediated Trk receptor signaling	0.40	1.28	0	0.08719759
Signaling mediated by p38-gamma and p38-delta	0.43	1.28	0.052208837	0.088682145
EPHB forward signaling	0.39	1.26	0	0.1028278
Role of Calcineurin-dependent NFAT signaling in lymphocytes	0.38	1.24	0	0.12479513
EPO signaling pathway	0.39	1.24	0.006	0.12763505
Validated nuclear estrogen receptor alpha network	0.39	1.23	0.005	0.1302045
amb2 Integrin signaling	0.38	1.23	0.001	0.13506135
N-cadherin signaling events	0.38	1.23	0.004	0.13909608
EGFR-dependent Endothelin signaling events	0.40	1.22	0.0960961	0.13963063
Canonical Wnt signaling pathway	0.40	1.22	0.076	0.14261521
Visual signal transduction: Rods	0.40	1.22	0.084084086	0.14717865
Signaling events mediated by Stem cell factor receptor (c-Kit)	0.37	1.21	0.004	0.16281918
Canonical NF-kappaB pathway	0.38	1.20	0.038	0.17255558
PAR4-mediated thrombin signaling events	0.43	1.19	0.22910216	0.19042686
Thromboxane A2 receptor signaling	0.37	1.19	0.011	0.19336416
Depleted				
EphrinA-EPHA pathway	-0.37	-1.10	0.28275862	0.11127982
LPA4-mediated signaling events	-0.18	-1.05	0.125	0.11279825

Supplementary Table 3. *Gene set enrichment analysis of autogenous interactions in NCI-PID pathways*. For each pathway we report: enrichment score (ES), Normalized enrichment score (NES), the nominal p-value (P-value), and the adjusted p-value (Q-value). The table lists enriched and depleted pathways with a False Discovery Rate (FDR) < 20%.

ENSP00000013034-ENST00000393196
ENSP00000044462-ENST00000413382
ENSP00000202773-ENST00000202773
ENSP00000202773-ENST00000424576
ENSP00000207437-ENST00000207437
ENSP00000222247-ENST00000222247
ENSP00000230050-ENST00000230050
ENSP00000233813-ENST00000449583
ENSP00000237500-ENST00000400175
ENSP00000237530-ENST00000373632
ENSP00000242577-ENST00000242577
ENSP00000242577-ENST00000392508
ENSP00000246554-ENST00000246554
ENSP00000251453-ENST00000251453
ENSP00000251453-ENST00000339471
ENSP00000252543-ENST00000394580
ENSP00000253452-ENST00000253452
ENSP00000256682-ENST00000447318
ENSP00000267884-ENST00000267884
ENSP00000267996-ENST00000403994
ENSP00000270142-ENST00000389995
ENSP00000272317-ENST00000402285
ENSP00000287038-ENST00000287038
ENSP00000294189-ENST00000492277
ENSP00000296417-ENST00000296417
ENSP00000301587-ENST00000344546
ENSP00000307786-ENST00000409409
ENSP00000307889-ENST00000467736
ENSP00000310275-ENST00000445560
ENSP00000311028-ENST00000312037
ENSP00000311028-ENST00000407193
ENSP00000322419-ENST00000321153
ENSP00000322421-ENST00000320868
ENSP00000325905-ENST00000446327
ENSP00000326031-ENST00000358239
ENSP00000333994-ENST00000335295
ENSP00000338345-ENST00000336904
ENSP00000338345-ENST00000345009
ENSP00000339095-ENST00000406376
ENSP00000341625-ENST00000299438
ENSP00000341625-ENST00000340533
ENSP00000346045-ENST00000330339
ENSP00000347271-ENST00000326199
ENSP00000347271-ENST00000344700
ENSP00000348918-ENST00000356524
ENSP00000349467-ENST00000447653
ENSP00000355778-ENST00000366815
ENSP00000356859-ENST00000367886
ENSP00000359910-ENST00000370861
ENSP00000359910-ENST00000370873
ENSP00000366124-ENST00000398409
ENSP00000372126-ENST00000382692
ENSP00000380156-ENST00000273223
ENSP00000386717-ENST00000409028
ENSP00000386717-ENST00000409320
ENSP00000386717-ENST00000409733
ENSP00000391944-ENST00000317198
ENSP00000419425-ENST00000355968
ENSP00000203407-ENST00000415995
ENSP00000229239-ENST00000474249
ENSP00000251595-ENST00000482565
ENSP00000272298-ENST00000484408
ENSP00000305260-ENST00000470354
ENSP00000307889-ENST00000399461
ENSP00000307889-ENST00000484610
ENSP00000307889-ENST00000491523
ENSP00000322421-ENST00000472694
ENSP00000327801-ENST00000473021
ENSP00000327801-ENST00000476482
ENSP00000327801-ENST00000478034
ENSP00000341625-ENST00000494131
ENSP00000345156-ENST00000479563
ENSP00000345344-ENST00000482054
ENSP00000346088-ENST00000471204
ENSP00000349142-ENST00000460820
ENSP00000363018-ENST00000467020
ENSP00000363018-ENST00000490335
ENSP00000381607-ENST00000464930
ENSP00000386717-ENST00000419276

Supplementary Table 4. *Protein-RNA interactions associated with low translation efficiency.* All the protein-RNA pairs showing an interaction propensity > 50, [protein]/[RNA]< 1 and protein abundance higher than 100 ppm are here reported. The high protein interaction propensity and poor translation efficiency indicates that a feedback loop mechanism is likely to occur in these cases.

Supplementary Figures

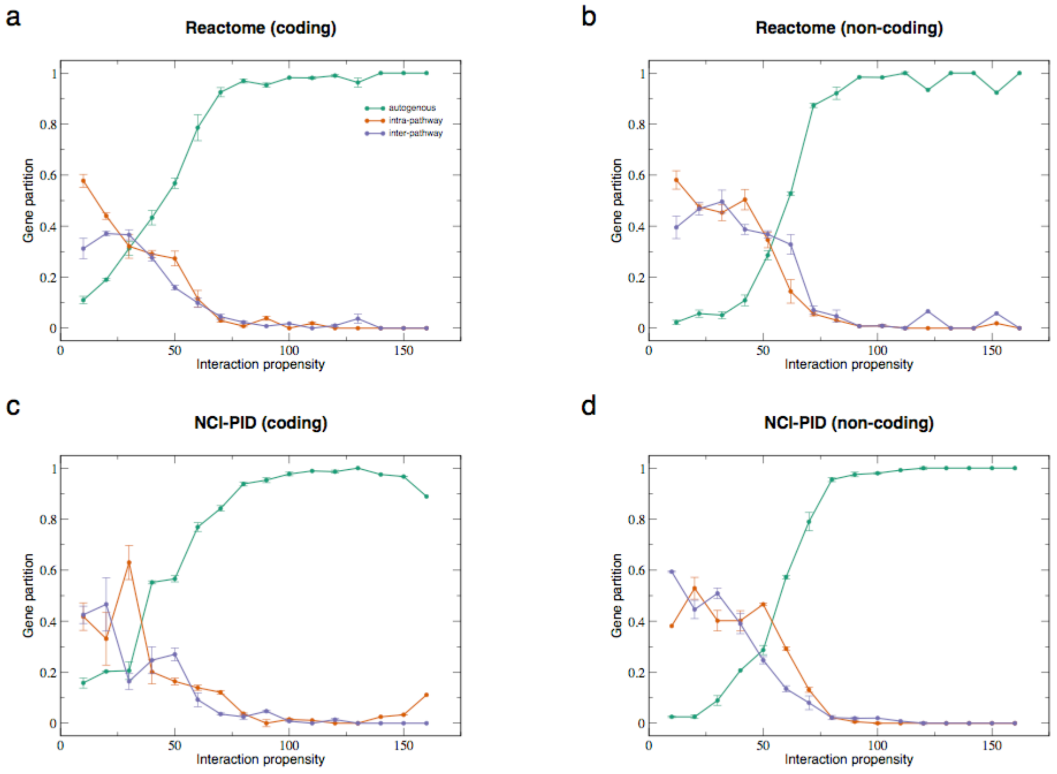

Supplementary Figure 1. *Autogenous associations are significantly enriched with respect to intra- and inter-pathway interactions in Reactome and NCI-PID databases*. This observation is valid for both coding (panel a for Reactome and panel c for NCI-PID pathways) and non-coding transcripts (panel b for Reactome and panel d for NCI-PID pathways). Autogenous, intra- and inter-pathway associations are represented with green, orange and blue lines, respectively. Note that panel a is the same as Figure 1b in the main text (mean and s.e.m. of bins are shown).

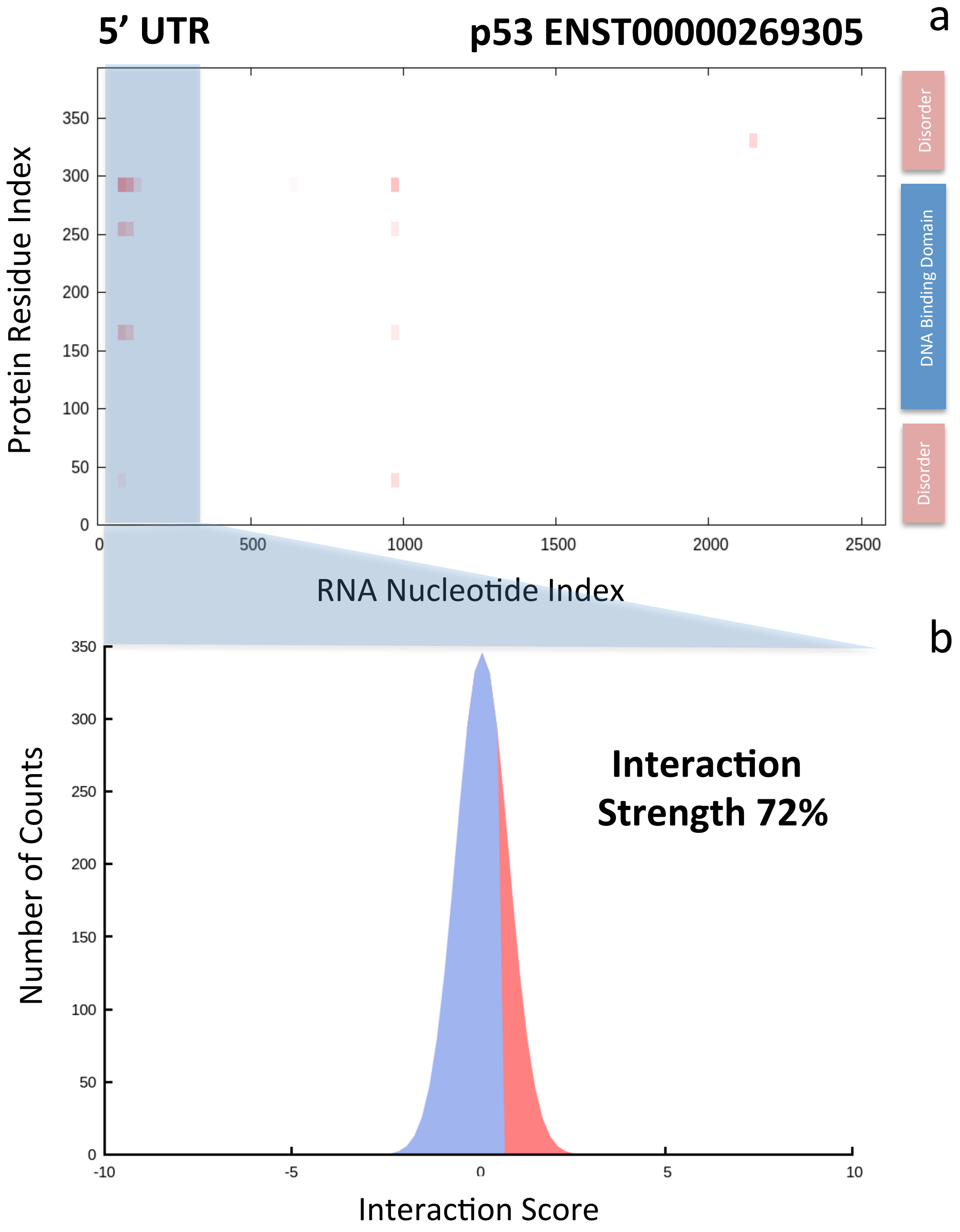

Supplementary Figure 2. *The autogenous interaction of p53.* a) In agreement with preliminary evidence, we predicted that human p53 interacts with its own transcript (ENST00000269305) at the 5' UTR. Notably, other regions in the coding sequence show propensity to interact with the mRNA, as shown in murine experiments. b) The interaction between the 5' UTR and p53 is strong (72%) with respect a pool of random protein-RNA associations.

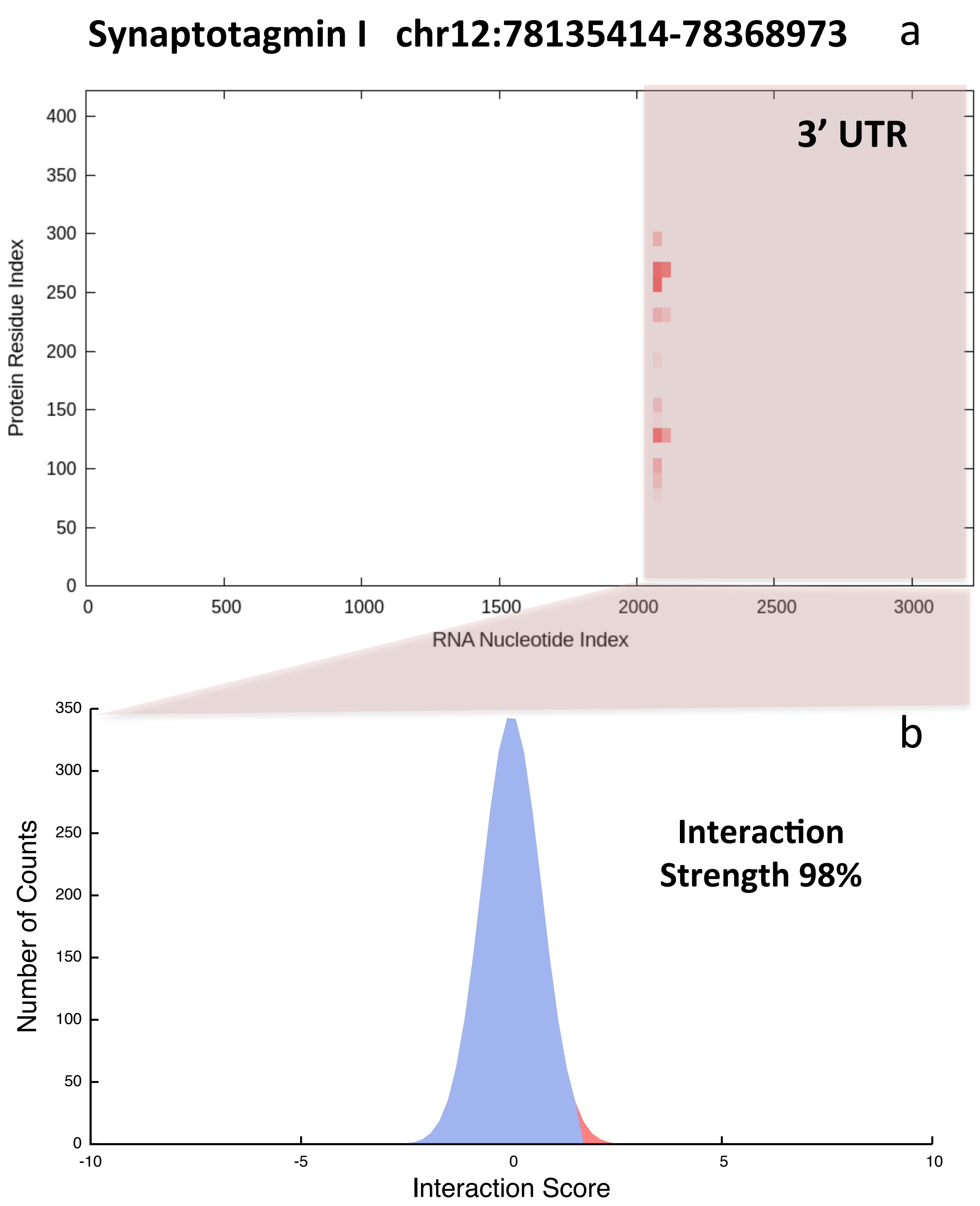

Supplementary Figure 3. *The autogenous interaction of human synaptotagmin-1.* a) In agreement with previous evidence, we predicted that human synaptotagmin-1 interacts with its own mRNA (chr12:78135414-78368973 of NCBI36/hg18) at the 3' UTR. b) The interaction between the 3' UTR and synaptotagmin-1 is strong (98%) with respect a pool of random protein-RNA associations, which indicates high specificity.

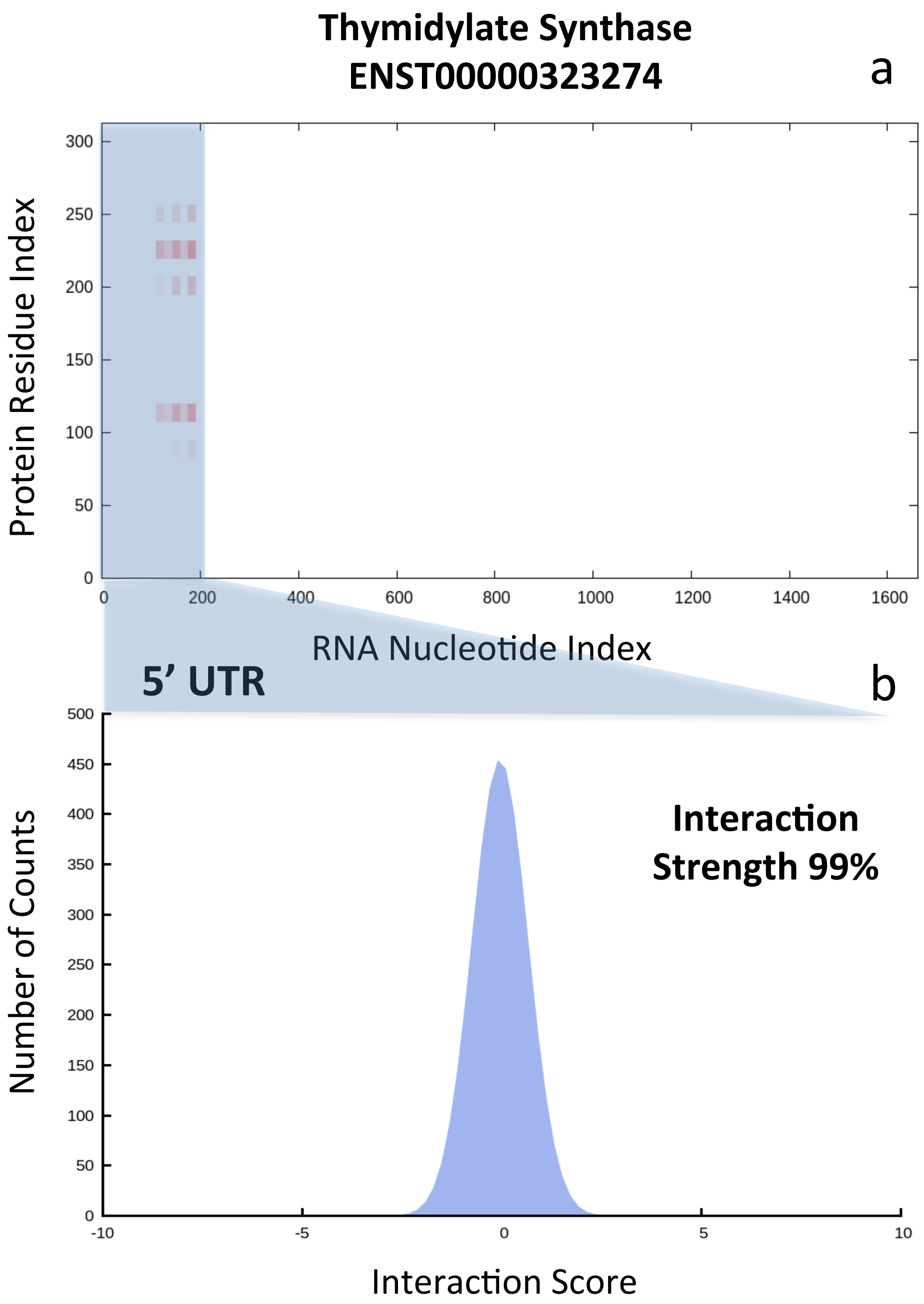

Supplementary Figure 4. *The autogenous interaction of human thymidylate synthase.* a) We predicted that human thymidylate synthase interacts with its own mRNA (ENST00000323274) at the 5' UTR, as shown in previous experiments. b) The interaction between the 5' UTR and thymidylate synthase is strong (99%) with respect a pool of random protein-RNA associations.

a

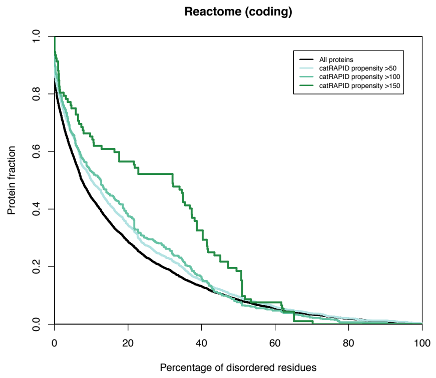

Protein set	Mean	Median	P-value (KS test)
All	16.23	7.40	-
Propensity > 50	18.53	10.10	2.0x10^{-11}
Propensity > 100	18.90	12.55	2.2x10^{-7}
Propensity > 150	26.82	32.10	1.3x10^{-9}

b

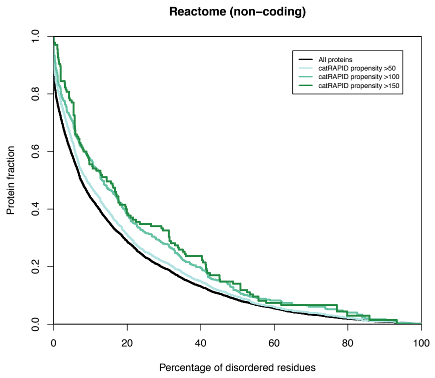

Protein set	Mean	Median	P-value (KS test)
All	16.23	7.40	-
Propensity > 50	17.73	9.00	5.1x10^{-8}
Propensity > 100	21.48	13.25	2.0x10^{-10}
Propensity > 150	22.72	14.40	6.9x10^{-5}

c

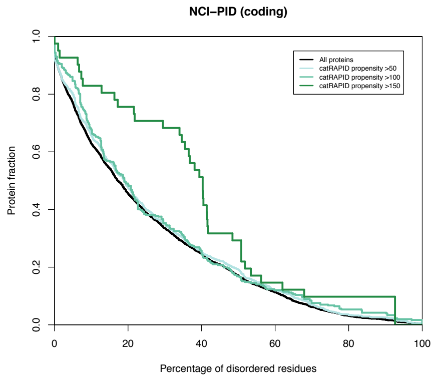

Protein set	Mean	Median	P-value (KS test)
All	25.03	17.20	-
Propensity > 50	26.20	19.00	0.11
Propensity > 100	26.97	19.00	0.0062
Propensity > 150	38.90	40.30	9.5x10^{-6}

d

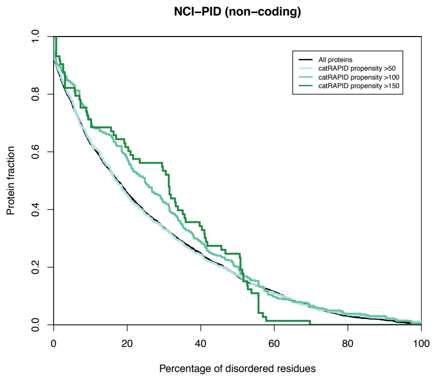

Protein set	Mean	Median	P-value (KS test)
All	25.03	17.20	-
Propensity > 50	25.07	17.00	1.00
Propensity > 100	28.94	24.80	1.2x10^{-5}
Propensity > 150	28.51	31.30	0.0019

Supplementary Figure 5. *Structural disorder in proteins involved in autogenous interactions in Reactome and NCI-PID pathways*. We observe an increase in disordered propensity from low to high protein-RNA interaction propensities (black line represents reference baseline) when considering interactions with either coding (panel a for Reactome and panel c for NCI-PID) or non-coding transcripts (panel b for Reactome and panel d for NCI-PID). We report the average and median values for all the proteins in each pathway database. The statistical significance was assessed with Kolmogorov–Smirnov test (KS test). Note that only at the lowest interaction propensity threshold (i.e. 50), the disorder propensity of NCI-PID proteins is not significantly different form the reference.

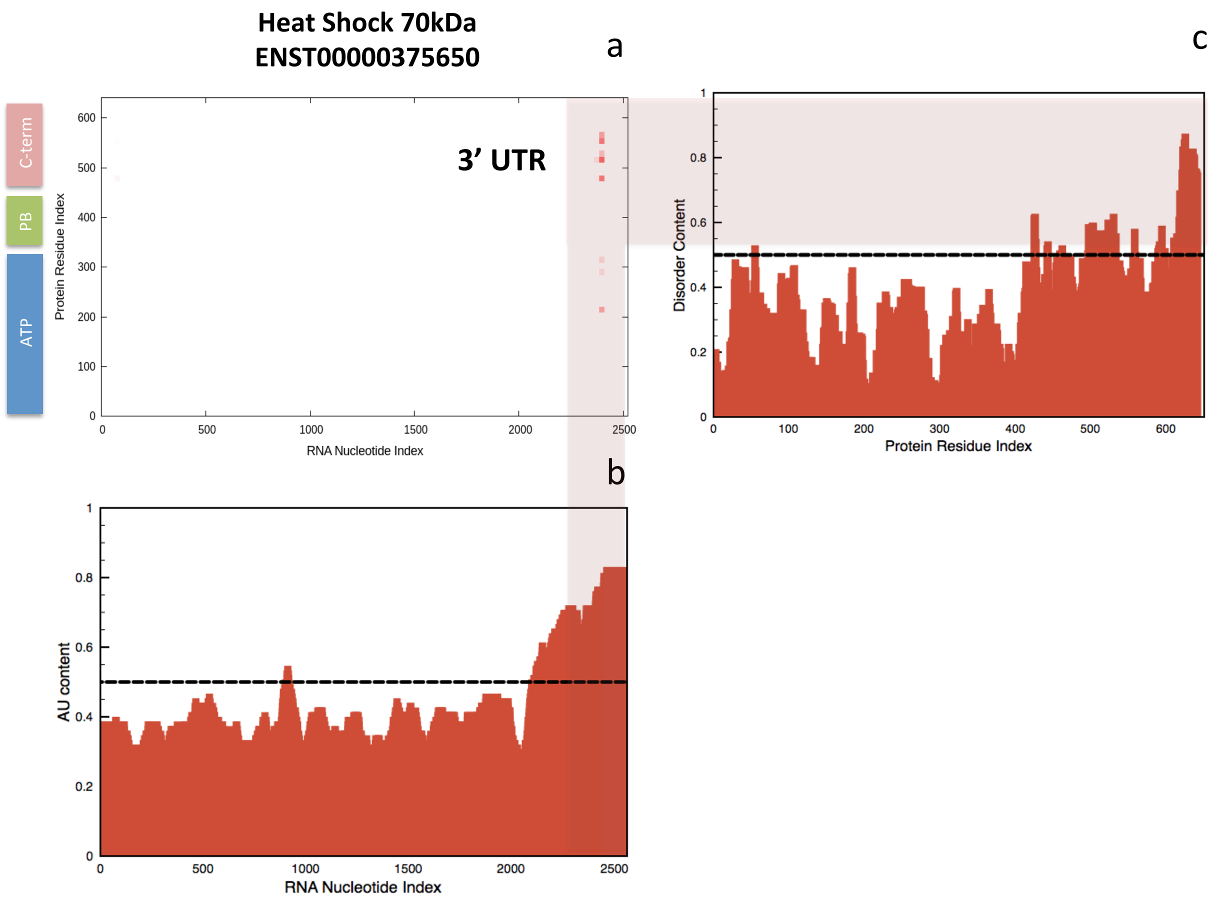

Supplementary Figure 6. *The autogenous interaction of Human Heat Shock 70kDa (HSP70).* a) In agreement with previous observations, we predicted that HSP70 interacts with its own mRNA (ENST00000375650) at the 3' UTR. b) HSP70 has a strong tendency to bind to AU-rich elements that accumulate at the 3' UTR. c) The N-terminal ATP domain is involved in the binding together with the disordered C-terminus, which is consistent with the fact that HSP70 RNA-binding affinity depends on the ATP domain but it is considerably reduced when the disordered region is removed (the disorder content was estimated with IUPred). Protein domains and disordered regions were taken from a recent study.

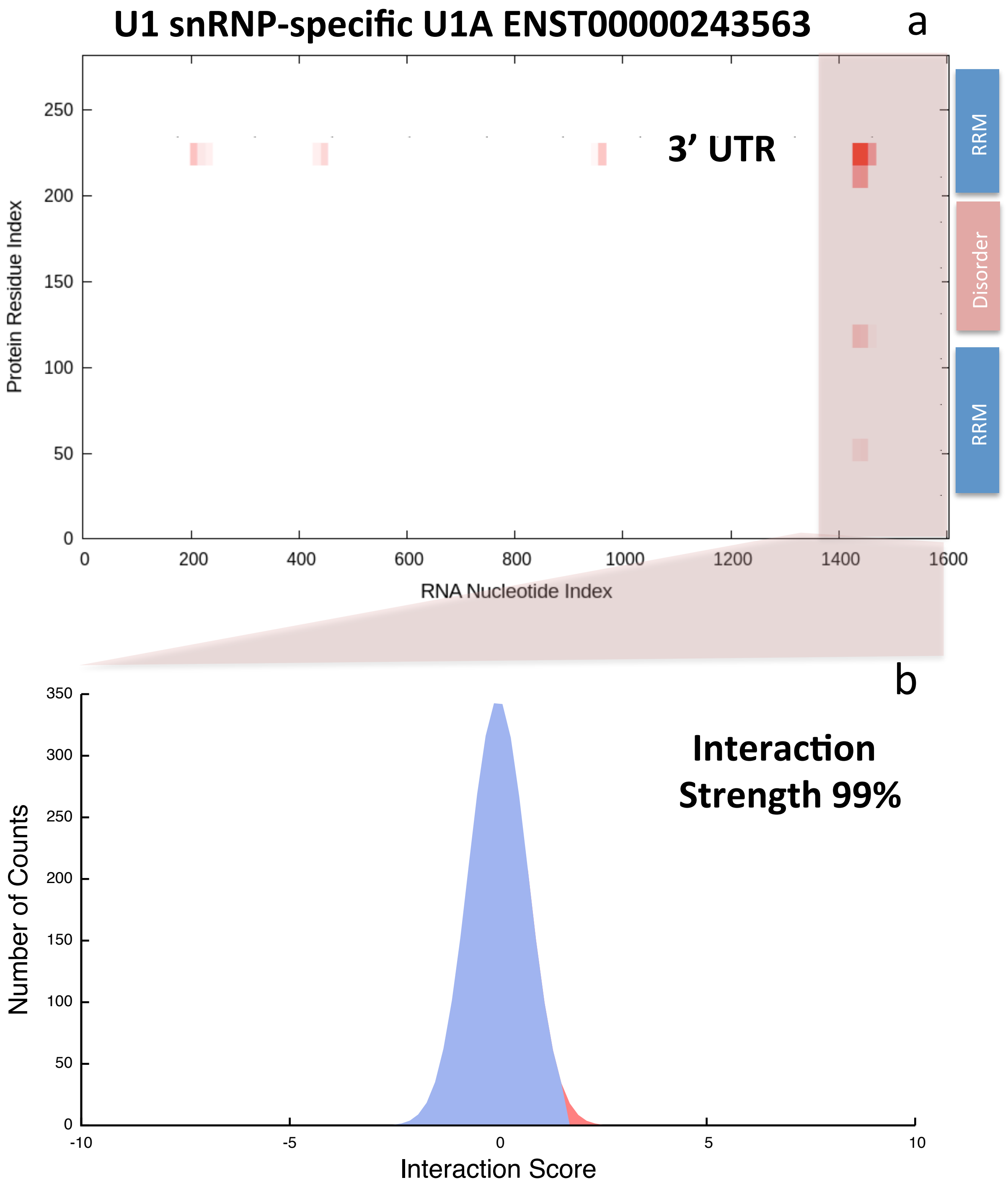

Supplementary Figure 7. *The autogenous interaction of human* small nuclear RiboNucleic Particle-specific (snRNP) U1A. a) In agreement with experimental evidence, we predicted that U1A synthase interacts with its own mRNA (ENST00000243563) at the 3’ UTR. b) The interaction between the 3’ UTR and U1A is strong (99%) with respect a pool of random protein-RNA associations (Methods), which indicates high specificity. The RNA binding domains and the disordered region are shown as reported in UniprotKB (entry P09012).

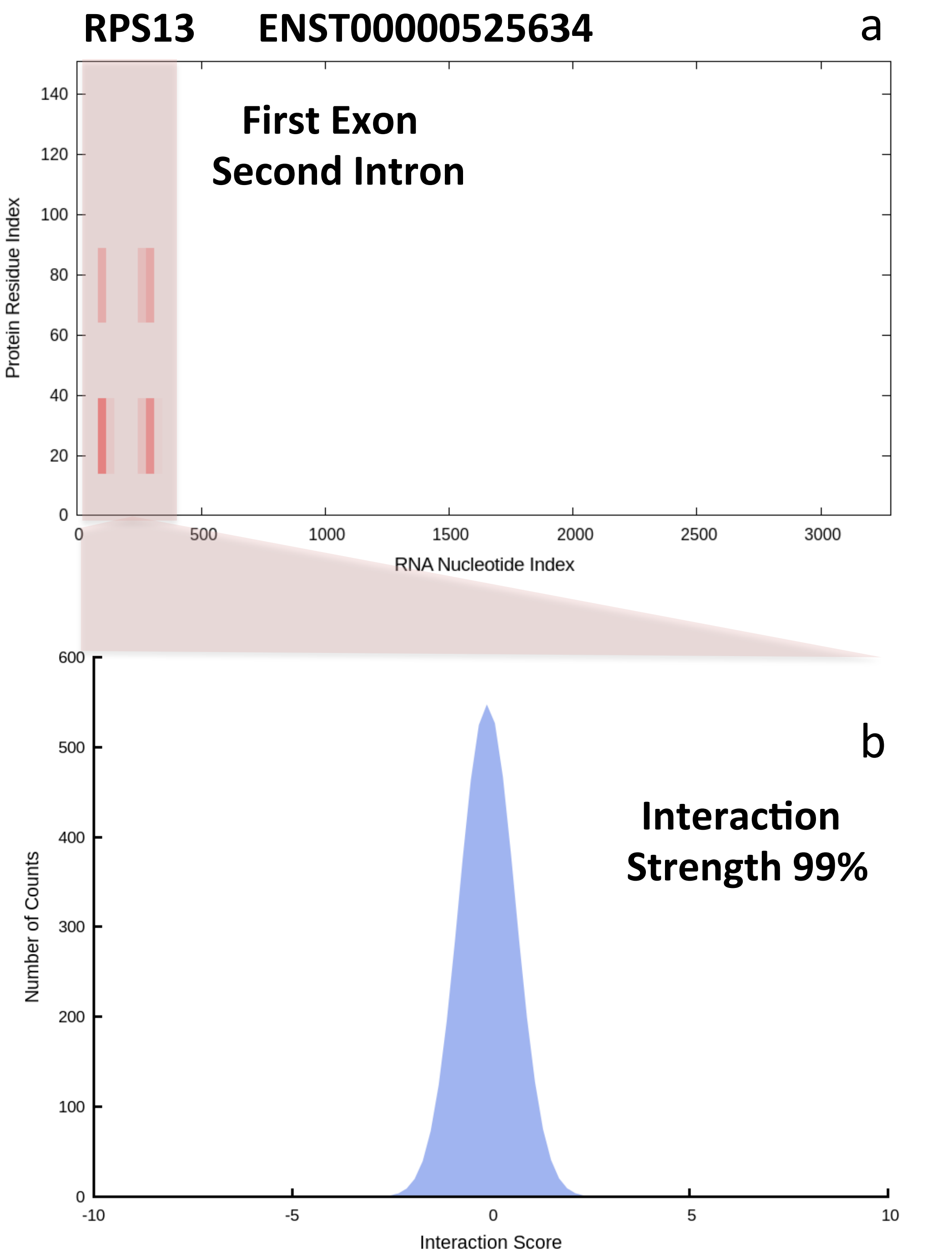

Supplementary Figure 8. *The autogenous interaction of human ribosomal protein S13.* a) *cat*RAPID identified the binding regions of S13 within the first and second exons (ENST00000525634), in agreement with experimental evidence. b) The interaction between the first and second exons with S13 is strong (99%) with respect a pool of random protein-RNA associations.

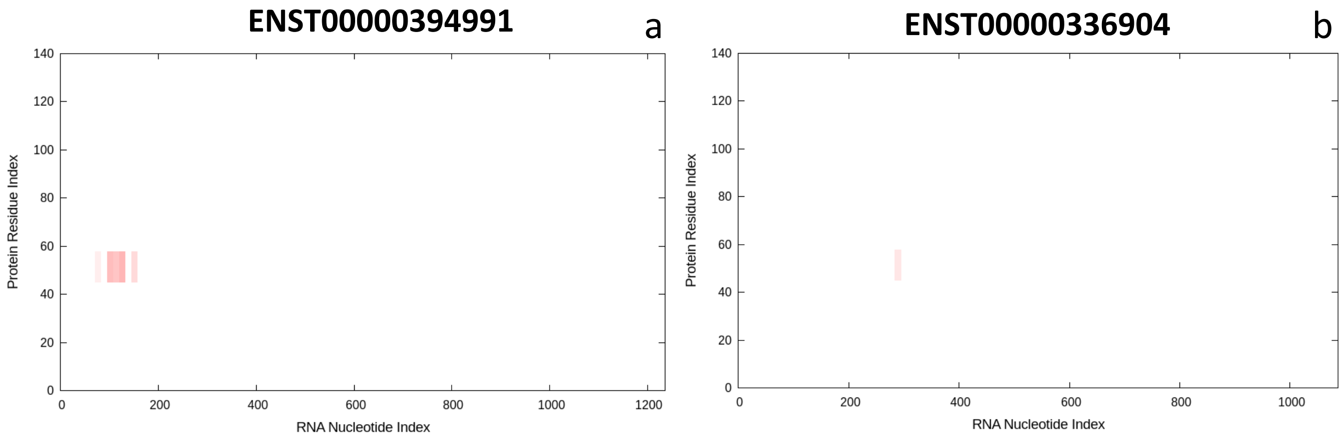

Supplementary Figure 9. *Interaction maps of* α*-synuclein with transcripts ENST00000394991 and ENST00000336904.* The binding sites were predicted to be in the 5' terminal regions. A lysine-rich region spanning residues 40-60 was predicted by *cat*RAPID to be involved in RNA recognition, which is consistent with previous results indicating an anion binding ability of the N-terminus.

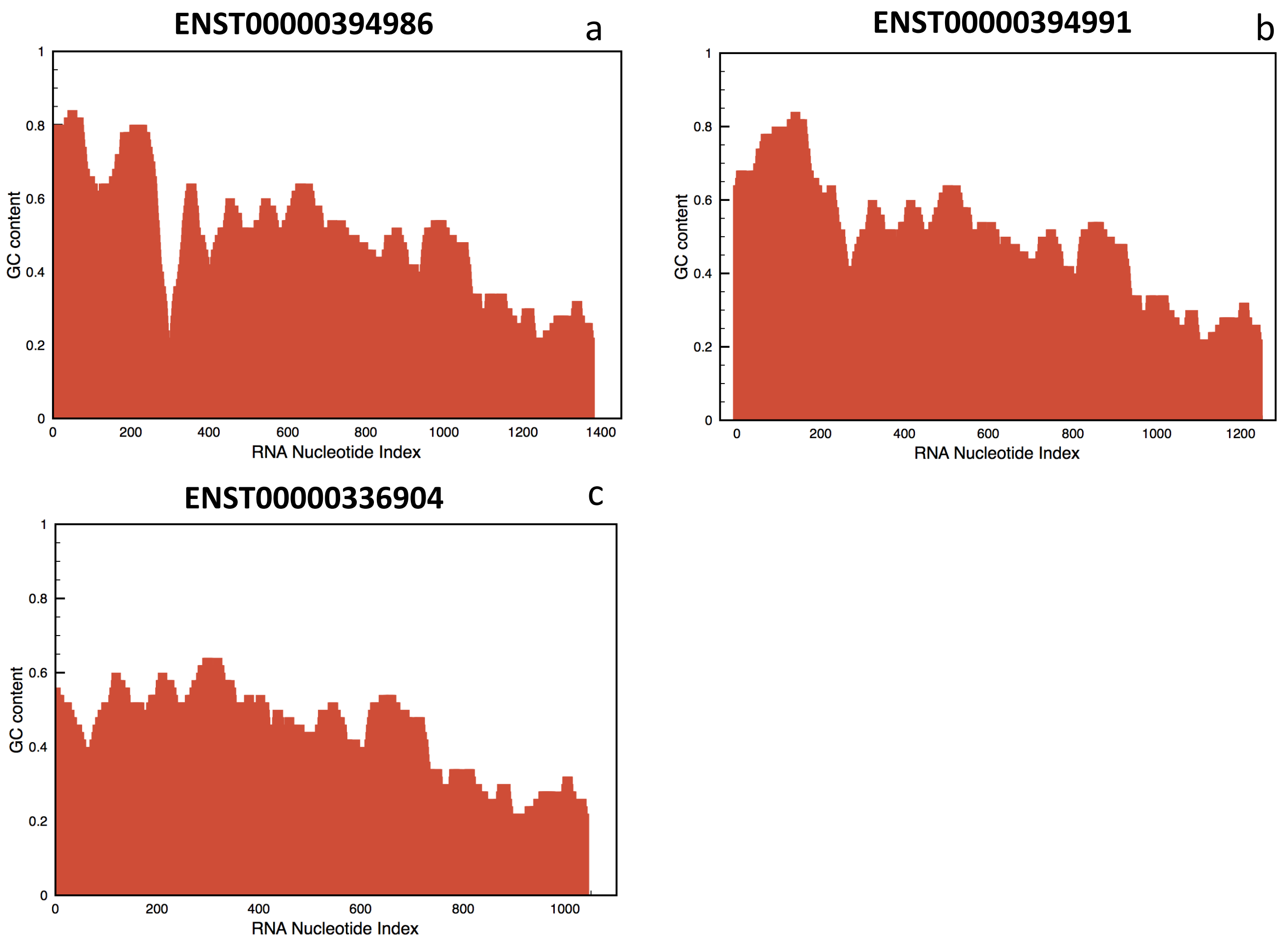

Supplementary Figure 10. *GC-content of* α*-synuclein transcripts ENST00000394986, ENST00000394991 and ENST00000336904.* The binding sites (Figure 5 and Supplementary Figure 9) were predicted to occur in GC-rich regions. Previous studies have shown that α-synuclein binds to DNA sequences enriched in GC content.
